# Antibiotic knowledge among ethnic minority groups in high-income countries: A mixed–methods systematic review

**DOI:** 10.1016/j.puhip.2025.100715

**Published:** 2025-12-23

**Authors:** Luisa Silva, Mayuri Gogoi, Zainab Lal, Paul Bird, Nisha George, Daniel Pan, Rebecca F. Baggaley, Pip Divall, Holly Reilly, Laura Nellums, Manish Pareek

**Affiliations:** a Department of Public Health and Epidemiology, University of Leicester, Leicester, UK; b Development Centre for Population Health, University of Leicester, Leicester, UK; c Department of Clinical Microbiology, University Hospitals of Leicester NHS Trust, Leicester, UK; d Department of Infectious Diseases and HIV Medicine, University Hospitals of Leicester NHS Trust, UK; e Leicester NIHR Biomedical Research Centre, Leicester, UK; f Institute of Health Informatics, University College London, London, UK; g Education Centre Library, University Hospitals of Leicester NHS Trust, Leicester, UK; h College of Population Health, Health Sciences Center, University of New Mexico, Albuquerque, NM, USA; i NIHR Applied Research Collaboration East Midlands (ARC EM), University of Leicester, Leicester, UK

**Keywords:** Antibiotic knowledge, Ethnicity, Ethnic minorities, Health inequalities, Antimicrobial resistance

## Abstract

**Objectives:**

Antimicrobial resistance (AMR) is a major global public health concern. Although low-income countries are disproportionately affected by AMR, certain underserved groups in high-income countries (HICs), such as migrants and ethnic minorities, disproportionately bear the burden of AMR. This may be driven by socio-cultural factors including differences in health literacy. This review aimed to investigate the level of antibiotic knowledge amongst different ethnic minority groups in HICs.

**Study design:**

This was a mixed-methods systematic literature review.

**Methods:**

We searched four databases (MEDLINE, EMBASE, the Cochrane library, CINAHL) to May 5, 2023, for primary studies on knowledge of antibiotics in different ethnic groups in HICs. We included studies in English using qualitative, quantitative and/or mixed-methods approaches and reporting on antibiotic knowledge by ethnicity. We used the convergent integrated approach for data synthesis and the Mixed-Methods Appraisal tool for quality assessment.

**Results:**

3935 articles were screened and 24 studies (17 quantitative, 5 qualitative, and 2 mixed-methods) were included, comprising 52778 participants from 8 countries (USA, UK, Australia, New Zealand, Netherlands, Greece, Sweden, Germany). Overall, participants from ethnic minority groups were able to identify common names of antibiotics and were aware of risks of antibiotics and side effects. However, participants thought antibiotics would treat viral-type illnesses. Ethnic minority groups generally had lower levels of knowledge compared to ethnic majority groups.

**Conclusions:**

Although ethnic minority communities possessed good levels of knowledge on certain aspects of antibiotics (e.g. being able to identify names of antibiotics), there were gaps in other areas (e.g. misperception that antibiotics are used for viral infections). The lower level of knowledge in ethnic minority groups compared to majority groups may be a contributing factor to health inequalities, which calls for co-designed, culturally competent, educational interventions.

## 1 Introduction

The use of antibiotics in the early 20th century marked a watershed moment in modern medicine as life expectancy rose considerably and mortality from infectious diseases reduced. However, the misuse (and overuse) of antibiotics has led to the emergence of antibiotic-resistant pathogens threatening lives and creating an ongoing antimicrobial resistance (AMR) crisis. AMR occurs when bacteria, viruses, fungi and parasites evolve to resist the effect of antimicrobial medicines, making infection harder or impossible to treat [1]. The global threat may have been further exacerbated by the COVID-19 pandemic [[2], [3], [4], [5]]. A recent publication from the Global Research on Antimicrobial Resistance (GRAM) Project reported that deaths directly attributable to bacterial AMR continue to rise and are projected to increase by nearly 70 % by 2050 [6]. While AMR poses a worldwide threat, its consequences are disproportionately felt by underserved populations, particularly those in low-resource settings [1]. In high-income countries (HICs), evidence suggests that ethnic minorities and migrant populations may be disproportionately affected by AMR [[7], [8], [9], [10], [11], [12]]. These disparities in AMR rates are influenced not only by clinical factors (such as age), but also by socio-cultural drivers including poor access to healthcare, low levels of antibiotic knowledge, and barriers related to language and cultural differences [13,14].

Despite the wide usage of antibiotics, global evidence has highlighted the association between public misconceptions about appropriate use and the causes of AMR. A large systematic review found widespread misunderstanding about AMR, with many people believing that it refers to individual immunity rather than microbial resistance, poses a low personal risk, or is a problem caused by others, with responsibility resting on health systems rather than the public [15]. A qualitative study conducted in Sweden highlighted the gap between the perceived personal risk of being affected by AMR and the seriousness of a future without antibiotics, suggesting challenges in how AMR prevention is communicated [16]. Other studies have found misconceptions about the use of antibiotic in viral infections [17,18]. If current trends continue, a future without effective antibiotics could mean that routine surgeries, childbirth, and even minor infections become life-threatening. AMR is projected to cause up to 10 million deaths annually by 2050, surpassing mortality from cancer and other major diseases, and potentially reversing decades of medical progress [19,20].

Moreover, it is widely accepted that there is often a lack of representation of ethnic minority groups, including migrants, in health services planning generally and in antimicrobial stewardship particularly [21,22]. Such inequities not only become drivers of AMR, but also create barriers to efforts aimed at addressing it, potentially leading to a lack of inclusivity in policies and decision-making related to AMR [13].

While studies have looked at disparities in AMR based on patient characteristics -including ethnicity - comprehensive evidence regarding differences in antibiotic knowledge and use among different ethnic groups is lacking. The aims of this review were to: (a) investigate the level of antibiotic knowledge amongst different ethnic groups in HICs; (b) explore if there are differences in antibiotic knowledge between ethnic ‘majority’ and ethnic ‘minority’ groups in HICs and; (c) propose possible implications of this evidence for future research and practice.

## 2 Methods

### 2.1 Study design

This is a mixed-methods systematic review of the literature to investigate the level of antibiotic knowledge amongst different ethnic minority groups in HICs.

### 2.2 Search strategy and selection criteria

PD searched four databases (MEDLINE and Embase via Ovid, the Cochrane CENTRAL, and CINAHL via EBSCOHost) from inception to May 5th, 2023 (see Supplementary Material for example of search strategy). We included primary studies published in English, using qualitative, quantitative and/or mixed-methods, conducted in all types of settings in HICs. The target ‘population’ were participants aged 18 and above. The ‘exposure’ was considered a particular ethnic minority group. ‘Ethnic minority’ was broadly defined as to include similar concept of ‘race’, as well as groups defined by migrant status or country of birth. For example, in the UK the term ‘ethnic minorities’ is used to refer to all ethnic groups except the White British group [23]. According to the 2021 Census for England and Wales, ethnic minority groups comprised 18 % of the overall population [24]. The ‘comparator’ were participants belonging to the ‘majority’ ethnic group, depending on the context where the study had taken place. Studies that investigated antibiotic knowledge in ethnic minority groups alone (i.e. not comparing them with the majority ethnic group) were still included, to increase the comprehensiveness of the review and ensure that all studies with ethnic minority groups were captured. This approach allowed for a broader understanding of the experiences and perspectives of these populations, even in the absence of direct comparative data with ethnic majority groups. The main ‘outcome’ was knowledge of antibiotics and antibiotic resistance. We excluded clinical case studies, case reports, systematic reviews and studies that did not include data on ethnicity/race, migration status or country of origin. We contacted corresponding study authors to obtain more information for some of the studies considered in the review. The review was registered on PROSPERO (CRD42023429367).

### 2.3 Study screening

All retrieved studies were imported into Rayyan for screening, and duplicates were removed. Three authors (LS, MG and ZL) independently screened all the titles and abstracts, and the full texts (where available) of all the identified studies. We resolved conflicts through discussion and when necessary, with the assistance of a fourth author (DP, RFB or MP).

### 2.4 Data extraction and analysis

Data extraction for each paper was independently done by two authors (LS, MG, ZL, PB or NG). Both entries were then matched for accuracy and rigour. The quality of each included study was independently assessed using the Mixed-Methods Appraisal tool (MMAT) version 2018 [25] by one of the reviewers (LS, MG, ZL), cross-checked by a second reviewer and any discrepancies were discussed with a third reviewer. An overall quality score for each study was reached by adding the ‘yes’ responses for that study and was represented as a percentage of the quality criteria that was met in that particular study [26] (see Supplementary Material). For mixed-methods studies, the overall score was based on the ‘yes’ responses in the lowest scoring component.

Our outcome of interest was antibiotic knowledge in different ethnic groups and we found that each study assessed knowledge differently accounting for heterogeneity in results. Studies assessed knowledge by checking if participants were able to identify antibiotics, awareness of risks and side-effects of antibiotics, recognising which conditions can be treated with antibiotics, knowledge about appropriate antibiotic use and/or knowledge about AMR. We report our convergent integrated analysis on each of these domains of knowledge, including a section on other socio-demographic variables, apart from ethnicity, which were found to be associated with antibiotic knowledge.

The quantitative studies in our review had significant heterogeneity in outcomes, which prevented us from conducting a meta-analysis of the data collected. We therefore opted to combine data from quantitative and qualitative studies using a convergent integrated approach, following updated Joanna Briggs methodological guidance for mixed-methods systematic reviews. The aim of this approach is to transform data into a mutually compatible format for integration [27]. Quantitative data were qualitised, i.e. numerical data were transformed into textual or narrative descriptions (example provided in Supplementary Material). Qualitative data did not go through any transformation process and was extracted directly from the studies. We then undertook a detailed examination of the assembled data and identified themes with similar meanings to produce integrated findings (example provided in Supplementary Material).

## 3 Results

**Fig. 1:**
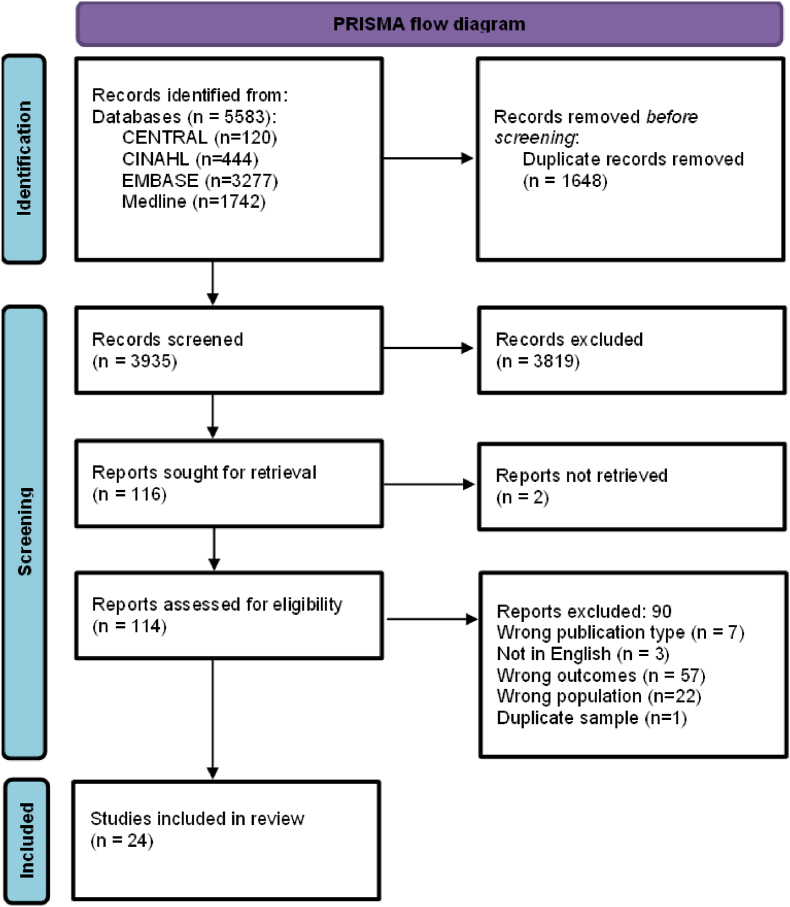
PRISMA flow diagram.

We identified 3935 unique records that met our inclusion criteria. 116 full-text articles were assessed for eligibility (one article could not be retrieved), of which 24 were included in the review (Fig. 1). The total sample size was 52,778, ranging from 23 to 21.617 participants. The target population and definitions for ‘ethnic minority groups’ varied by study, some used definitions according to ethnicity (n = 17) and others by country of origin (n = 7). Participants were recruited either from healthcare settings (primary care or hospital) (n = 2), or from the community (i.e. general population, including online) (n = 20) or a combination of both (n = 2).

Data collection methods comprised interviews, focus groups, surveys/questionnaires or a combination of these. In terms of quality assessment, nearly half of included studies (11/24, 46 %) were considered of high methodological quality (MMAT score of 80 % or above). There was however significant variation in study quality with six studies scoring quite low (0–40 % on the MMAT) and seven achieving average results (60 % on MMAT). An overview of the studies’ characteristics is provided in Table 1 and additional information is included as Supplementary Material.

**Table 1:** Overview of included studies (n = 24).

Characteristic		Number of studies, n = 24 (%)		Number of study participants, N = 52778 (%)	
Study design	Quantitative	17	(71 %)	52348	(99.2 %)
	Qualitative	5	(21 %)	278	(0.53 %)
	Mixed-methods	2	(8 %)	152	(0.29 %)
Country	USA	10	(40 %)	12945	(24 %)
	UK	5	(20 %)	11807	(22 %)
	New Zealand	3	(12 %)	455	(0.86 %)
	Australia	3	(12 %)	448	0.84 %)
	Netherlands	2	(8 %)	21681	(41 %)
	Greece	1	(4 %)	5312	(10 %)
	Multinational	1	(4 %)	130	(0.24 %)
Setting	Healthcare setting (primary care or hospital)	2	(8 %)	767	(1.45 %)
	Community setting (general population, including online)	20	(83 %)	51954	(98.4 %)
	Healthcare setting and community	2	(8 %)	57	(0.11 %)
Data collection method	Questionnaires/survey	17	(71 %)	52348	(99.2 %)
	Interviews	3	(12.5 %)	84	(0.16 %)
	Focus groups	1	(4 %)	64	(0.12 %)
	Focus groups + Interviews	1	(4 %)	130	(0.25 %)
	Focus groups + questionnaires	1	(4 %)	27	(0.05 %)
	Interviews + questionnaires	1	(4 %)	125	(0.24 %)

		**Number of studies, n = 24 (%)**		**Ethnic minority group categories**
Ethnic minority group categories used, by country	USA	10	(40 %)	Asian Americans, Asian, Hawaiian/Pacific Islanders, Black/African American, Hispano-Latinos, Arabic/Arabic Americans, American Indian Mixed/other
	UK	5	(20 %)	Migrants from African countries (including Zimbabwe, Sudan, Cameroon, DR Congo, Uganda, Ivory Coast), India, Iran, China, Poland, Pakistan, India; Non-White participants (compared with White British), Other White background, Asian, Black Caribbean, Black African, Chinese/other
	New Zealand	3	(12 %)	Indian, Egyptian, Korean backgrounds, Indigenous Māori, Samoans
	Australia	3	(12 %)	Chinese migrants, ‘diverse ethnic communities’ (1st generation migrant communities)
	The Netherlands	2	(8 %)	Migrants from Suriname, Ghana, Morocco, Turkey, Cape Verde and Syria
	Greece	1	(4 %)	Immigrants (compared to non-immigrants)
	Germany, Sweden, the Netherlands	1	(4 %)	Turkish migrants

Ethnic differences in knowledge have been reported for each domain separately. However, five studies did not present disaggregated data by each antibiotic knowledge domain and instead reported a composite outcome. Panagakou et al., 2012 (Greece), for example, used an overall antibiotic knowledge score based on four questions (related to ‘recognising which conditions can be treated with antibiotics’ and ‘knowledge of antimicrobial resistance’), and immigrants performed worse compared to non-immigrant participants [28]. Similarly, Kuzujanakis et al., 2003 (USA) reported White race to be associated with higher antibiotic knowledge [29]; McNulty et al., 2007 (UK) found Asian or Black Caribbean respondents gave more incorrect responses to the antibiotic statements as compared to White British respondents [30]; Pieper et al., 2020 (USA) reported African American participants had significantly lower antibiotic knowledge scores than non-African Americans and Lescure et al., 2022 (The Netherlands) did not find significant differences between immigrants and native Dutch participants [31,32].

Below we report the results for each domain of antibiotic knowledge and a summary of the integrated findings is also provided in Table 2.

1. Recognising names of antibiotics

**Table 2:** Summary of integrated findings related to antibiotic knowledge.

Integrated finding	Contributing studies, n Sample size across the studies, N	Qualitative data	Qualitised data
1.Recognising names of antibiotics	Quantitative studies = 5 Mixed methods = 2 N = 6444	**Confusion between antibiotics and other types of medications:** Norris 2009 [34]: One respondent, speaking of her nephew with epilepsy: *Interviewer*: “Do you remember what medication he was on?” *Participant*: “He was definitely on some sort of antibiotics but I have no idea what they were, I will have to ask my sister. I think it was two lots of different ones”.	**Good knowledge of names of antibiotics, particularly penicillins, although some confusion between antibiotics and other types of medications:** Kandakai 1996 [33]: Majority able to identify penicillin (89 %) and amoxicillin (63 %) as antibiotics, but less than half able to identify tetracycline (43 %); these medications were correctly identified as not being antibiotics by a proportion of participants - darvon (57 %), acetaminophen (71 %), muscle relaxant (41 %), tylenol (34 %) and aspirin (28 %). Norris 2009 [34]: Just over half (54 %) of participants correctly identified antibiotics from a list of different medications; vast majority able to identify amoxicillin and augmetin. Hu 2015 [36]: Vast majority (79 %) able to identify amoxicillin. **Variable knowledge of names of antibiotics:** Larson 2006 [35]: Being able to identify antibiotics from a list of commonly used drugs varied considerably between participants (12–92 %). **Ethnic differences in ability of identify names of antibiotics:** Panagakou 2012 [28]: Majority of people were able to identify antibiotics from a list of medications; non-immigrants performed better than immigrants (6.7 % vs 35.9 % respectively answered incorrectly on this question). Norris 2010 [37]: Most Indian (67 %) and Egyptian (83 %) respondents correctly identified amoxicillin and augmentin as antibiotics, but a range of other commonly used medicines were also identified as antibiotics by the respondents.

2.Awareness of risks and side effects of antibiotics	Qualitative studies = 4 Quantitative studies = 7 Mixed methods = 1 N = 19213	**Antibiotics seen as strong medications, which can cause side effects:** Lescure 2022 [32]: “Yes, currently, all mothers know exactly that antibiotics are not normal medicines, that they are not paracetamol. They also have disadvantages, and it is not good if they are always accessible.” Whittaker 2019 [44]: “It's my opinion because, when virus attack you, they just damage your organ outside. But, when you use some antibiotic, they damage your, inside your organs.” Other participant felt that “some antibiotics very strong, you know. If the person is weak, is, she doesn't have enough energy to take these things, so how can she cope?”. Hika 2022 [41]: Participants expressed concerns about effect of antibiotics on their bodies “[antibiotics are] more harmful than anything else.". Some also believed that “if you take too much, you could damage your [body] system more”. Lindenmeyer 2016 [42]: Certain participants (particularly those from Iran and China) and some African countries believed that antibiotics are ‘strong medicine’ with powerful effect on the individual, including strong side effects.	**Good knowledge of possible side effects and allergies:** Kandakai 1996 [33]: Majority of participants able to identify signs of allergic reactions - nausea (75 %), rash (65 %), swelling (63 %), fever (56 %) and half also identified diarrhoea. Majority were also able to exclude other symptoms as not being associated with allergies - dizziness (81 %), shakes (75 %), pain (69 %). Hu 2015 [36]: Majority (79 %) of participants were aware of possible side effects. **Limited knowledge regarding risks and side effects:** McNulty 2007 [30]: Significant lack of knowledge among participants regarding harmful effects of antibiotics. Larson 2006 [35]: Only some participants (36 %) were aware that antibiotics can causes risks to health. McNulty 2022 [40]: Although a large portion of participants (68 %) agreed that ‘antibiotics kill bacteria living in our gut’, some (39 %) also agreed that ‘antibiotics do not affect other bacteria in our bodies’. **No differences between ethnic groups regarding knowledge of side effects:** Broniatowski 2018 [39]: There were no differences between ethnic groups regarding side effects of antibiotics. Watkins 2015 [43]: Knowledge of antibiotic side effects was generally comparable between Hispanic consumers and all consumers.

3. Recognising which conditions can be treated with antibiotics	Qualitative studies = 3 Quantitative studies = 14 Mixed methods = 2 N = 38667	**Vague descriptions of antibiotics and variable knowledge regarding indication of antibiotics for bacterial rather than viral infections:** Whittaker 2019 [44]: When asked to describe an antibiotic one participant replied that it was ‘medicine’ used “sometimes [for] fever, sometimes cough, sometimes pain”. Lescure 2022 [32]: Some participants knew that antibiotics are only effective against bacterial infections; others thought that antibiotics are necessary in treating viral infections. Hika 2022 [41]: Participants were not able to distinguish between a bacterial and viral infection.	**Varied knowledge regarding conditions treated with antibiotics, including misperception of their use in viral infections:** Norris 2009 [34]: Majority (81 %) believed antibiotics were useful for colds and flu and the majority also thought it would prevent cold and flus from getting worse (68 %). Larson 2006 [35]: Majority thought that antibiotics could help cure a cold (56 %), prevent an ear infection (72 %), and treating bacteria and viruses (56 %). Pieper 2020 [31]: Many respondents believed that can cure colds and flu (10/26); more than half of the respondents (15/26) reported that antibiotics are good for treating infections caused by viruses. Corbett 2005 [46]: Less than half (43 %) of the participants replied correctly that antibiotics are not needed to treat cold. McNulty 2007 [30]: Many participants (37.4 %) did not know antibiotics do not work on most cough on colds. McNulty 2019 [17]: Majority of participants (72–83 %) understood antibiotics are used to treat bacterial infections, while a minority (35–43 %) thought they are used to treat viral infections. Kuzujanakis 2003 [29]: Many respondents did not know that antibiotics are helpful only in bacterial infections (34 %) and that viruses cause most colds, coughs, and flu (24 %). McNulty 2022 [40]: Only about a fifth of respondents (23 %) mentioned the word ‘bacteria’ specifically when asked about what is an antibiotic. In contrast most respondents (81 %) selected bacteria from a list when asked ‘which conditions can be treated effectively by antibiotics?’ Kandakai 1996 [33]: Most people were able to identify conditions commonly treated with antibiotics - sexually transmitted infections (83 %), bacterial infections (82 %) and also when antibiotics are not indicated - viral infections (92 %), flu (81 %), common cold (77 %), pain (57 %). Alden 2006 [45]: Average to good knowledge regarding appropriate antibiotic use in treating upper respiratory tract infections. Mason 2018 [47]: Majority of participants in both affluent and deprived areas demonstrated correct knowledge on conditions treated with antibiotics. Hu 2015 [36]: Majority aware antibiotics used for urinary tract infections (61 %), others thought antibiotics were used for fever (35 %), cough (21 %), diarrhoea (14 %), sore throat/827 %), abdominal pain (3 %) and toothache (18 %). Schwartz 2017 [49]: Many participants understood antibiotics are used to cure bacterial infections (37.9 %), while a small proportion believed antibiotics are effective for viral infections (9.6 %) or both (25.1 %). Norris 2010 [37]: Majority of participants (73.3 %) believed that antibiotics killed bacteria; some respondents also said that antibiotics strengthen the immune system (24.3 %), kill viruses (19.7 %), and heal illness (24.0 %); more than a quarter (28.3 %) incorrectly believed antibiotics were useful for colds and flu, and another quarter (26.3 %) were unsure. Schuts 2019 [48]: Non-Dutch groups were less knowledgeable about antibiotics than Dutch when asked about antibiotic treatment during influenza-like illness, pneumonia, fever, sore throat and bronchitis. Broniatowski 2018 [39]: African-American participants (ED sample) had lower results on the items related to the use of antibiotics against bacteria (and not viruses), compared to other ethnicities included in the study.

4. Knowledge about antibiotic use	Qualitative studies = 3 Quantitative studies = 3 Mixed methods = 1 N = 1332	**Variable knowledge of use of antibiotics:** Lindenmeyer 2016 [42]: “I had frostbites which became septic but I couldn't have antibiotics because in this country, it takes a long time until you are given antibiotics, yet back home in our countries, as long as there is sepsis or infection, even high temperature antibiotics are given. And with us when we come from abroad we would be used to antibiotics. […] I couldn't [retrain as a nurse] because my fingers were septic and I was afraid again to as I have to be aware of infecting other people”. Hika 2022 [41]: Participants indicated lack of knowledge about antibiotic usage. **Ethnic minority group had poorer knowledge than majority group:** Westerling 2020 [50]: Reduced understanding of antibiotic rational use in Turkish participants compared to host population.	**Good knowledge of appropriate use of antibiotics:** Pieper 2020 [31]: Most people (23/26) said that it is not ok to use leftover antibiotics if you are sick or have a wound or sore. Similar number of respondents (22/26) also said that it is not OK to stop using antibiotics when a person starts feeling better and/or symptoms have stopped (eg, fever) and that antibiotics cannot be stored and used as needed at a later date. Mason 2018 [47]: Majority of participants in both affluent and deprived areas demonstrated correct knowledge on antibiotic usage. However, many more participants in affluent areas displayed better understanding by taking antibiotics only when prescribed (99 % vs 58 % respectively) and by never stopping them with improvement of symptoms (85 % vs. 34 % respectively). Larson 2006 [35]: Some thought that antibiotics should be stopped as soon as the person feels better (24 %). Schwartz 2017 [49]: Majority of participants (87.3 %) preferred avoiding antibiotics unless advised otherwise by their doctor; those who preferred to not take antibiotics were three times more likely to have correct knowledge about antibiotics compared to those who preferred taking them.

5. Knowledge about antimicrobial resistance	Qualitative studies = 2 Quantitative studies = 6 Mixed methods = 1 N = 12425	**Varied knowledge regarding AMR:** Lescure 2022 [32]: “When I stop the antibiotic treatment too quickly, bacteria in my body will become stronger and the next time these bacteria will not be defeated by the same antibiotics. So, the antibiotic will become a sweet for the bacteria. The bacteria will say, ‘come to me, I will eat you, I will not die from you”. Whittaker 2019 [44]: [When asked about causes of AMR] on participant mentioned “the weather or because of the environment? I'm not sure”.	**Good awareness of AMR and contributing factors:** Larson 2006 [35]: Many participants were aware that some germs are becoming harder to treat with antibiotics (44 %) and that bacteria can become resistant to antibiotics if the dose (60 %) or length (48 %) of course is inadequate. Hu 2015 [36]: Vast majority were aware of antibiotic resistance (84 %). Pieper 2020 [31]: A large number of respondents (69.2 %) have heard of AMR. McNulty 2022 [40]: The vast majority of participants recognised that ‘overuse’ or ‘taking any’ antibiotics caused resistance (90 %); Half of the respondents (50 %) said that healthy people carry antibiotic resistant bacteria. **Varied knowledge regarding AMR amongst different ethnic groups:** Corbett 2005 [46]: Majority (80 %) of Non-Hispanic Whites strongly agreed with the statement that ‘‘some germs are becoming harder to treat with antibiotics’’. Of the Hispanics, many (76 %) of those who took the survey in English reported strong agreement, as compared to the Spanish speaking Hispanics (49 %). Norris 2010 [37]: Awareness of resistance varied between groups and was high amongst Egyptians (55 %) and Koreans (36 %) but was particularly low amongst Indians (12 %). Watkins 2015 [43]: Hispanic consumers were less aware of potential dangers of antibiotic use, such as antibiotics becoming less effective after their use (antibiotic resistance).

6. Other variables associated with antibiotic knowledge: 6.1 Formal education 6.2 Socioeconomic status 6.3 Gender 6.4 Age	Qualitative studies = 1 Quantitative studies = 9 N: Formal education = 4149 Socioeconomic status = 2413 Gender = 29500 Age = 22723	Westerling 2020 [50]: **Lower antibiotic knowledge linked with lower socioeconomical and educational level.**	**Higher formal education and socioeconomic status associated with better antibiotic knowledge:** Pieper 2020 [31]: Higher formal education associated with higher levels of antibiotics knowledge. Alden 2006 [45]: Higher formal education was associated with higher levels of antibiotic knowledge. Kuzujanakis 2003 [29]: Antibiotic knowledge was associated with increased parental age and education. McNulty 2019 [17]: Higher social grade and higher qualifications were strongly positively associated with knowledge of antibiotics and antimicrobial resistance. Mason 2018 [47]: Many participants in affluent areas (81 %) displayed better understanding that antibiotics do not help cure common cold, cough and flu, compared to those in deprived areas (45 %). **Education and occupation not associated with antibiotic knowledge:** Landers 2010 [38]: Education and occupation were not associated with significant differences in knowledge of antibiotics (specifically identifying names of antibiotics). **Women and older participants had better antibiotic knowledge** McNulty 2007 [30]: Women (23.2 %) were less likely than men (25.7 %) to give incorrect responses about antibiotic knowledge. Schwartz 2017 [49]: Women had better knowledge of antibiotics than men. Schuts 2019 [48]: Participants under 25 years-old had less knowledge than all other age groups; women had more knowledge than men.

Seven out of the 24 studies measured knowledge by asking participants to identify names of antibiotics [28,[33], [34], [35], [36], [37], [38]]. Participants from ethnic minority backgrounds were overall able to recognise common names of antibiotics, although other medications were at times incorrectly identified as antibiotics). In one study, the number of participants able to correctly identify antibiotics from a list of commonly used medications ranged from 12 % to 92 % [35]. Panagakou et al., 2012 (Greece) looked at differences between immigrant and non-immigrant participants in Greece and found that migrants performed worse in this area (35.9 % of immigrants answered incorrectly a selected question in this topic, compared to 6.7 % in the of non-immigrants’ group) [28].

2. Awareness of risks and side effects of antibiotics

Twelve studies looked at ethnic minority participants' awareness of risks of antibiotics, including side effects and allergies [30,33,35,36,[39], [40], [41], [42], [43], [44]]. Results for this integrated finding were varied – one study found a lack of knowledge about the potential harmful effects of antibiotics. For example, McNulty et al. (2007) (UK) [30] found limited awareness among participants, with 43 % unaware that antibiotics can kill bacteria that normally live on the skin and in the gut. Two studies reported limited knowledge among participants about side-effects. Larson et al. (2006) [35] found that only 36 % of participants were aware that antibiotics cause risks to health, while Pieper et al. (2020) (USA) observed similar trends [31]. In contrast, two studies reported good knowledge of side-effects [33,36]. For example, Hu and Wang (2015) (Australia) [36] found that 79 % of the participants were aware of potential side effects of antibiotics. Additionally, four studies reported that antibiotics are considered a ‘strong medicine’ with a harmful effect on the body [32,41,42,44]. There were no differences between ethnic groups in the two studies that specifically looked at this variable [39,43].

3. Recognising which conditions can be treated with antibiotics

The knowledge of the reasons for prescribing antibiotics was examined in 19 out of 24 studies [17,29,37,39,41,44,49]. In ten studies, participants from ethnic minority backgrounds correctly identified when antibiotics may be used [17,32,33,[35], [36], [37],^40^,^45^,^47^,^49^] but the majority of the studies showed that participants thought viral diseases, such as cold or flu, could also be treated with antibiotics [e.g. Refs. ([35], [37])]. Regarding differences between ethnic groups, one study concluded that an ethnic minority group (African-American participants) scored lower on the items related to the use of antibiotics against bacteria (and not viruses), compared to other ethnicities included in the research [39]. The study by Alden et al., 2006 (USA) found that some ethnic minority groups (Filipinos and Hawaiian/Pacific Islanders) had lower antibiotic knowledge than White participants [45]. In Schuts et al., 2019 (The Netherlands), non-Dutch groups were less knowledgeable about antibiotics than Dutch respondents [48]. Schwartz et al., 2017 (USA) found White respondents were significantly more likely to have correct knowledge as compared to those of any other race [49].

4. Knowledge about appropriate antibiotic use

Seven studies provided information on ethnic minority participants’ knowledge about the correct use of antibiotics (e.g. finishing course of antibiotics) [31,35,41,42,47,49,50]. There was, however, heterogeneity in results from the different studies: two studies reported lack of knowledge about antibiotics usage [35,41], whereas two other studies found participants had a better understanding about this [31,49]. There was limited information about ethnic differences, with only one study [50] reporting that an ethnic minority group (in this case Turkish participants) had reduced understanding about rational use of antibiotics compared to the host population (Germany, the Netherlands and Sweden). Lindenmeyer et al., 2016 (UK) found that participants from African countries and India felt that they needed to take antibiotics quickly to cure illness and prevent future infection [42].

5. Knowledge about antimicrobial resistance

Knowledge of AMR amongst ethnic minority groups was explored in nine studies [31,32,35,37,40,43,44,46]. In the majority of these studies (n = 8) [31,32,[35], [36], [37],^40^,^43^,^44^,^46^], participants had an understanding of AMR and the factors contributing to it (e.g. Larson et al., 2006 (USA) [35] reported that many participants were aware that bacteria can become resistant to antibiotics if the dose (60 %) or length (48 %) of antibiotic course was inadequate). One qualitative study showed that participants had vague or limited knowledge of AMR, even amongst those infected with AMR infections [44]. Additionally, in Watkins et al.’s study 2015 (USA), the ethnic minority group (Hispanic group) was less aware of the risk of antibiotic resistance compared to other groups in the study [43].

6. Other variables associated with antibiotic knowledge:
  6.1. Formal education
    Formal education was found to be associated with better knowledge of antibiotics in 5 out of the 6 studies that looked at this variable [17,29,31,45,46]. Only one study found that education did not significantly contribute to differences in knowledge [38].

6.2. Socioeconomic status
  There were limited data available on this variable, but two studies concluded that higher socio-economic status led to better knowledge of antibiotics [17,50], whereas Landers et al., 2010 (USA) did not find any significant differences in knowledge based on participants’ occupation [38]. Finally, in Mason et al., 2018 (UK) ethnicity did not influence knowledge about antibiotic usage, but participants in affluent areas had better understanding of use of antibiotics compared to more deprived areas [47].

6.3. Gender
  Four studies provided data on whether antibiotic knowledge differed by gender. In three of these studies, women were found to have better overall knowledge of antibiotics compared to men [30,48,49], however, one study did not find differences in knowledge between male and female participants [31].

6.4. Age
  This variable was considered in two of the included studies and in both, younger participants had overall less knowledge compared to older participants [29,48]. However, Schuts et al., 2019 (The Netherlands) also looked at knowledge between 1st and 2nd generation migrants within the ethnic minority groups and concluded that the first generation (older) participants performed worse than second generation groups [48].

## 4 Discussion

We undertook a systematic review to investigate antibiotic knowledge amongst different ethnic groups in HICs. Overall, participants from ethnic minority groups were able to identify common names of antibiotics and were aware of potential risks and side effects of their use. However, there was confusion regarding the indication of antibiotics, such as the perception that these medications were used to treat viral-type illnesses. While some studies reported good knowledge of antibiotic use and awareness of AMR, our review highlighted that ethnic minority groups in HICs generally had lower levels of antibiotic knowledge compared to ethnic majority groups. In addition to ethnicity, several other variables were found to be related to antibiotic knowledge, including formal education and socioeconomic status, gender and age.

A significant finding of our review was that participants perceived antibiotics as being effective against viral infections, which aligns with previous research [17,51]. This could potentially be because, in many languages spoken by ethnic minority communities in high-income countries, the terms for bacterial and viral infections are indistinct [44]. Our findings suggest varied levels of antibiotic knowledge in ethnic minority groups, depending on the specific aspects being studied, but more importantly, it helps consolidate the growing evidence on the issue of health inequalities, since ethnic minority groups demonstrated lower levels of antibiotic knowledge when compared to ethnic majority groups.

However, it is important to acknowledge that not all ethnic minority groups consistently show lower levels of antibiotic knowledge, and several factors may explain this variation. For instance, acculturation and length of residence in the host country can influence health literacy, with longer-term residents often having better access to health information and services [52]. Additionally, community-level health education initiatives, particularly those delivered through trusted cultural or religious institutions, may enhance antibiotic knowledge in certain groups [53,54].

This review reinforces that antibiotic knowledge is influenced by a range of social, cultural, economic and contextual determinants. Importantly, having more antibiotic knowledge does not always directly correlate with better antibiotic consumption behaviours, as some studies have shown that higher levels of antibiotic knowledge are linked with more inappropriate use of antibiotics [55]. Inappropriate antibiotic use is shaped by complex human behaviour at multiple levels. While antibiotic knowledge is a key predictor, actual behaviour around antibiotics is more nuanced, influenced by habits, environmental cues, and social contexts. As Kelly points out in his research on behaviour change, behaviours that may appear irrational from a health perspective often have logical explanations when considered within the broader context of individuals’ lives [56]. This complexity must be acknowledged by policymakers when designing antimicrobial stewardship campaigns. Rather than viewing inappropriate antibiotic consumption purely as a knowledge deficit, efforts should focus on the broader social and behavioural factors that drive misuse.

To our knowledge, this is the first systematic review to look at differences in antibiotic knowledge in ethnic minority groups in HICs. The main strength of this review lies in its confirmation of the need to improve antibiotic knowledge in these communities. In the context of health promotion in disadvantaged groups, the relevance of assessing knowledge of antibiotics is that improving knowledge, may change attitudes towards these widely used (and misused) medications and this can in turn have implications for future behaviour, i.e. contribute towards better practices around antibiotic use [57]. This study has some limitations. Firstly, the concept of antibiotic knowledge was defined in different ways in the included studies, which made it hard to compare different studies. However, the different interpretations of antibiotic knowledge helped define the main integrated findings. Additionally, many studies looked at both aspects of antibiotic knowledge and antibiotic use, which made data extraction challenging at times and required several team discussions to clarify what was more directly related to our outcome of interest. Many of the studies are considered of low quality (particularly some quantitative studies) and with small samples sizes, which may limit the power and generalizability of the findings.

In conclusion, our review found that, overall, ethnic minority groups in high-income countries had lower levels of antibiotic knowledge compared to ethnic majority groups. This highlights the need for increased awareness of antibiotics amongst ethnic minority groups by tailoring culturally sensitive public health campaigns for different ethnicities and linguistic groups promoting rational use, including school-based educational interventions. With the increasing burden of antimicrobial-resistant infections in HICs, particularly among ethnic minority and migrant populations, there is an urgent need for robust evidence to clarify how antibiotic knowledge – or the lack thereof – contributes to antibiotic (mis)use in underserved communities.

## 5 What this study adds

• Provides the first mixed-methods systematic review synthesising evidence on antibiotic knowledge specifically among ethnic minority groups in high-income countries.
• Demonstrates that ethnic minority groups generally have lower levels of antibiotic knowledge than majority groups across several domains (e.g., indications, resistance, appropriate use).
• Highlights that antibiotic knowledge is shaped by intersecting socio-demographic factors - including education, socioeconomic status, age, and gender - emphasising the complexity of inequalities influencing antimicrobial stewardship.

6 **Implications for policy and practice**

• Tailored, culturally and linguistically sensitive public health campaigns are needed to improve antibiotic knowledge and promote appropriate use among diverse ethnic communities.
• Policies to strengthen antimicrobial stewardship should address broader social determinants - such as access to healthcare, health literacy, and socioeconomic barriers - rather than focusing solely on knowledge deficits.
• Community-based and school-based educational interventions, co-designed with ethnic minority groups, can support more equitable engagement with AMR prevention and enhance the effectiveness of stewardship programmes.

## Ethical statement

No ethical approval was required as this review used existing published data.

## Funding

This study was supported by the 10.13039/501100000272National Institute for Health and Care Research (NIHR) Applied Research Collaboration East Midlands (ARC EM) and Leicester NIHR Biomedical Research Centre (BRC). The funder of the study had no role in study design, data collection, data analysis, data interpretation, or writing of the report.

DN was supported by an NIHR Doctoral Research Fellowship (NIHR302338). RFB was supported by an NIHR Advanced Fellowship (NIHR302494). The views expressed are those of the authors and not necessarily those of the NIHR or the Department of Health and Social Care.

## Declaration of competing interest

The authors declare that they have no known competing financial interests or personal relationships that could have appeared to influence the work reported in this paper.
