# Supplementary material for: Antibiotic knowledge among ethnic minority groups in high-income countries: A mixed–methods systematic review
Source: Public Health Pract (Oxf). 2025 Dec 23;11:100715. doi: 10.1016/j.puhip.2025.100715 (PMC12818139; doi:10.1016/j.puhip.2025.100715)
Supplement: Multimedia component 1 [file mmc1.docx]

**Supplementary data**

**Antibiotic knowledge among ethnic minority groups in high-income countries: a mixed–methods systematic review**

Silva *et al*.

| **Contents** | **Page** |
| --- | --- |
| Search strategy for Ovid Medline | 2 |
| Characteristics of included studies and outcomes reported | 5 |
| Quality assessment | 14 |
| List of excluded studies at full-text screening stage with reasons for exclusion | 16 |
| Example of data analysis process | 24 |

1. **Search strategy for Ovid MEDLINE(R) ALL <1946 to May 09, 2023>**

- 1 (ethnicity or ethnic).mp. [mp=title, book title, abstract, original title, name of substance word, subject heading word, floating sub-heading word, keyword heading word, organism supplementary concept word, protocol supplementary concept word, rare disease supplementary concept word, unique identifier, synonyms, population supplementary concept word, anatomy supplementary concept word] 208509
- 2 Minority Groups/ 17726
- 3 continental population groups/ 25404
- 4 hispanic americans/ 33532
- 5 african continental ancestry group/ 40362
- 6 American Native Continental Ancestry Group/ 1298
- 7 Asian Continental Ancestry Group/ 73382
- 8 European Continental Ancestry Group/ 71389
- 9 Oceanic Ancestry Group/ 0
- 10 African Americans/ 61571
- 11 Arabs/ 5497
- 12 Asian Americans/ 9015
- 13 (multi?cultural or multi cultural or cross?cultural or cross cultural or trans?cultural or transcultural).mp. [mp=title, book title, abstract, original title, name of substance word, subject heading word, floating sub-heading word, keyword heading word, organism supplementary concept word, protocol supplementary concept word, rare disease supplementary concept word, unique identifier, synonyms, population supplementary concept word, anatomy supplementary concept word] 45339
- 14 (BAME or minority or minorities).mp. [mp=title, book title, abstract, original title, name of substance word, subject heading word, floating sub-heading word, keyword heading word, organism supplementary concept word, protocol supplementary concept word, rare disease supplementary concept word, unique identifier, synonyms, population supplementary concept word, anatomy supplementary concept word] 102951
- 15 exp Population Groups/ 328536
- 16 "transients and migrants"/ 14155
- 17 migrant*.ti,ab,kw,kf. 25227
- 18 expatriate*.ti,ab,kw,kf. 1356
- 19 asylum.ti,ab,kw,kf. 4567
- 20 foreign-born.ti,ab,kw,kf. 4067
- 21 indigenous.ti,ab,kw,kf. or Indigenous Peoples/ 42273
- 22 Ethnic Groups/ 72637
- 23 refugee*.ti,ab,kw,kf. or Refugees/ 18287
- 24 aboriginal*.ti,ab,kw,kf. 10478
- 25 "country of birth".ti,ab,kw,kf. 2373
- 26 ((Minorit* or Mixed) adj3 (ethnic* or race)).ti,ab,kw,kf. 21255
- 27 (Arab* or Africa* or Afro* or Asian or Bangladesh* or Black or Caribbean or Chinese or India* or Irish or (Mixed adj other) or Multi-rac* or Pakistan* or Roma or traveller* or traveler* or Gyps* or Gips*).ti,ab,kw,kf. 1263200
- 28 (multicultural or "multi cultural" or multiethnic or "multi ethnic" or multiracial or "multi racial" or crosscultural or "cross cultural" or "trans cultural" or transcultural or biracial).ti,ab,kw,kf. 35725
- 29 immigrant*.ti,ab,kw,kf. 30175
- 30 (Ethnic* or racial or race or racis* or "people of color" or "people of colour" or POC or "BME" or "BAME").ti,ab,kw,kf. 311235
- 31 or/1-30 1753154
- 32 exp Anti-Bacterial Agents/ 812022
- 33 Drug Resistance, Bacterial/ 47971
- 34 Drug Resistance, Microbial/ 61050
- 35 (antibiotic* or anti-bacterial* or anti-microbial* or antibacterial* or antimicrobial*).ti,ab,kw,kf. 642237
- 36 or/32-35 1153965
- 37 31 and 36 50163
- 38 exp Health Knowledge, Attitudes, Practice/ or Patient Medication Knowledge/ 126433
- 39 health literacy/ 9048
- 40 health education/ 63547
- 41 Patient Education as Topic/ 88262
- 42 ((health or medication or medicine) adj2 (literacy or literate or knowledge or attitude* or education)).mp. [mp=title, book title, abstract, original title, name of substance word, subject heading word, floating sub-heading word, keyword heading word, organism supplementary concept word, protocol supplementary concept word, rare disease supplementary concept word, unique identifier, synonyms, population supplementary concept word, anatomy supplementary concept word] 430248
- 43 ((antibiotic* or medication* or medicine* or anti-bacterial* or anti-microbial* or antibacterial* or antimicrobial*) adj6 (knowledge* or belief* or percep* or perceive* or understand* or aware* or attitud*)).ti,ab,kw,kf. 35811
- 44 Qualitative Research/ 80919
- 45 qualitative.ti. 69367
- 46 or/38-45 614340
- 47 37 and 46 1742

**2. Characteristics of included studies and outcomes reported (including overall MMAT score)**

**Table X**

| **First author Year**  **Country** | **Participants** | **Outcome of interest** | **Measurement tool/Method** | **Main findings** | **MMAT %** |
| --- | --- | --- | --- | --- | --- |
| **Quantitative studies (n = 17)** | | | | | |
| Alden 2006, USA | Asian Americans,  Hawaiian/Pacific Islanders and Whites in a major metropolitan area | Knowledge regarding appropriate antibiotic use in treating upper respiratory infections (URI) | Survey - 8-item scale to assess when antibiotic were an appropriate treatment & second scale 5 agree/disagree statements | Some ethnic minority groups (Filipinos and Hawaiian/Pacific Islanders) had lower antibiotic knowledge than Whites. Other ethnic groups had intermediate results. Average to good knowledge regarding appropriate antibiotic use in treating URI. Higher formal education associated with better antibiotic knowledge. | 40% |
| Broniatowski 2018, USA | Online sample from general population; patients from emergency department (ED); ethnicity: White, Black/African American, Asian, Mixed/other, Hispanic; *also included healthcare providers but not included in the review* | Knowledge about antibiotics, side effects and use for Strep throat infection | Survey - online or paper in ED sample | African-American participants (ED sample) had lower results on the items related to the use of antibiotics against bacteria (and not viruses), compared to other ethnicities included in the study; Side effects of antibiotics - no differences in knowledge between ethnic groups; Strep throat infection - no differences in knowledge regarding indication of antibiotics | 60% |
| Corbett 2005, USA | Adult member of the household within the general population who were non-Hispanic whites (NHW) or Hispanic–Latino | Knowledge about appropriate antibiotic  use | Survey | For knowledge about antibiotic use for colds, correct responses were given by more than half of Non-Hispanic Whites (NHW) (53%), in contrast with 19% of Hispanics (P < 0.001); Majority (80%) of NHW strongly agreed with the statement that ‘‘some germs are becoming harder to treat with antibiotics’’. Of the Hispanics, many (76%) of those who took the survey in English reported strong agreement, as compared to the Spanish-speaking Hispanics (49%) | 60% |
| Hu 2015*, Australia | Chinese migrants in Australia | Identify antibiotics, indication, side effects and knowledge of antibiotic resistance | Web-based survey | Majority able to recognise amoxillin (79%) and were aware of side effects (79%) and antibiotic resistance (84%). Varied knowledge regarding indication of antibiotics. | 40% |
| Kandakai 1996, USA | African Americans | Identify antibiotics, allergic reactions/side effects and indications of antibiotics | Open-ended elicitation questionnaire | Majority able to identify penicillin (89%) and amoxicillin (63%) as antibiotics, but less than half able to identify tetracycline (43%). Majority able to identify signs of allergic reactions and conditions commonly treated with antibiotics. | 60% |
| Kuzujanakis 2003, USA | Parents with a child <6 years old; Ethnicities: Caucasian, African American, Hispanic, Asian, Other | Indication of antibiotics. | Survey | Many respondents did not know that antibiotics are helpful only in bacterial  infections (34%) and that viruses cause most colds, coughs, and flu (24%). Antibiotic knowledge was associated with white race, increased parental age and education, having more than 1 child, and receipt of media information on resistance. | 80% |
| Landers 2010, USA | Latino population in the northern Manhattan. | Identify antibiotic. | Survey | Medications were correctly identified in nearly half (49.3%) of instances. Non antibiotics were properly identified in nearly two thirds (62%]) of responses although antibiotics were properly identified in only about a third (34%) of the times. Education, months in the trial, and occupation were not associated with significant differences in identification scores. | 60% |
| Mason 2016, UK | Members of the public and community pharmacists in affluent and deprived areas. White and Non-White considered. | Indication antibiotics, antibiotic use. | Survey | Majority of participants in both affluent and deprived areas demonstrated correct knowledge on antibiotic usage. However, many participants in affluent areas (81%) displayed better understanding that antibiotics do not help cure common cold, cough and flu, compared to those in deprived areas (45%). Vast majority of participants in affluent areas showed better understanding of antibiotic usage compared to those in deprived areas, by taking antibiotics only when prescribed (99% vs 58%). Majority of participants (85%) in affluent areas showed better understanding of antibiotic usage compared to deprived areas (34%), by never stopping them with improvement of symptoms. Ethnicity did not influence knowledge about antibiotic usage | 60% |
| McNulty 2007, UK | Public households; Ethnic groups: White British, other White background, Asian, black Caribbean, black African, Chinese/other | Indication of antibiotics, side effects, knowledge of antibiotic use. | Survey | Significant lack of knowledge among participants about effectiveness and harmful effects of antibiotics. Many participants (37.4%) did not know antibiotics do not work on most cough on colds and 43% did not know that antibiotics kill bacteria that normally live on the skin and in the gut.  Asian (37%) and Black Caribbean (34%) respondents gave more incorrect responses about antibiotic knowledge compared to White British participants (23.7%). Women (23.2%) were less likely than men (25.7%) to give incorrect responses about antibiotic knowledge. In women, better knowledge of antibiotics was associated with sharing them with someone for whom they were not prescribed. | 100% |
| McNulty 2019, UK | Public households; Ethnic groups: White, BAME (Black, Asian and Minority Ethnic) | Indication of antibiotics, antibiotic resistance. | Survey | Majority of participants (72-83%) understood antibiotics are used to treat bacterial infections, while a minority (35-43%) thought they are used to treat viral infections. Participants from ethnic minority groups had less knowledge about antibiotic resistance compared to White ethnic groups. Higher social grade and higher qualifications were strongly positively associated with knowledge of antibiotics and antimicrobial resistance.  Adults in households with children or who had visited a doctor or pharmacy in the preceding 12 months were more knowledgeable than adults with no children in households and those who had not been to a doctor/ pharmacy in the last 12 months. | 80% |
| McNulty 2022, UK | Public households. Ethnic groups: White, BAME (Black, Asian and Minority Ethnic) | Indication of antibiotics, antibiotic use, antibiotic resistance. | Surveys | Most respondents (81%) selected bacteria from a list when asked ‘which conditions can be treated effectively by antibiotics?’  About one third incorrectly stated antibiotics effectively treat viral or fungal infections.  Many respondents did not know that antibiotics work for ‘the majority of urine infections’(25%) or ‘make heart operations and cancer treatments safer’ (43%).  Majority of respondents (84%) said that coughs, colds and sore throats get better on their own without the need for antibiotics’.  The vast majority of participants recognised that ‘overuse’ or ‘taking any’ antibiotics caused resistance. A large portion of participants (68%) agreed that ‘antibiotics kill bacteria living in our gut’. Knowledge that antibiotics treat bacterial infections is significantly higher in white versus ethnic minority groups. | 80% |
| Norris 2010, New Zealand | Adults who belong to either Indian, Egyptian or Korean ethnic backgrounds, over the age of 18 years. | Indication of antibiotics, identify antibiotics, side effects. | Questionnaire | Majority of participants (73.3%) believed that antibiotics killed bacteria.  Some respondents also said that antibiotics strengthen the immune system (24.3%), kill viruses (19.7%), and heal illness (24.0%).  All incorrect answers (i.e. relieving pain, strengthening the immune system, and killing viruses) were particularly common amongst Korean respondents (27%, 42% and 42%, respectively). Few Korean respondents (20%) correctly identified the role of antibiotics.Most Indian (67%) and Egyptian (83%) respondents correctly identified amoxil/amoxicillin and Augmentin as antibiotics, but a range of other commonly used medicines were also identified as antibiotics by the respondents. Misconceptions were particularly common amongst the Koreans.  Nearly half (41%) of Koreans incorrectly thought that Panadol (acetaminophen) was an antibiotic. Of the total sample, more than a quarter (28.3%) incorrectly believed antibiotics were useful for colds and flu, and another quarter (26.3%) were unsure.  Groups differed significantly in their responses about antibiotic need: Egyptians were the most likely to think that antibiotics were useful for colds and flu (45%), followed by Koreans (29%) and Indians (11%).  When asked, more than half (61.3%) of the total sample said that antibiotics can have ‘‘bad effects’’. The most common ‘‘bad effect’’ pointed to was ‘‘germs get used to them and get harder to kill’’, i.e. antibiotic resistance (34.3% of the total sample).  Awareness of resistance varied between groups and was high amongst Egyptians (55%) and Koreans (36%) but was particularly low amongst Indians (12%). | 20% |
| Panagakou 2012, Greece | Parents of a child/children aged between 5 and 6 years. Immigrants and non-immigrants. | Identify antibiotic, indication antibiotics. | Survey | Immigrants performed worse compared to non-immigrants participants when asked to identify names of antibiotics.  Immigrants also performed worse on the antibiotic knowledge questions (always used in fever; viral conditions; knowledge about AMR with inappropriate use). | 100% |
| Pieper 2020, USA | Adults who reported a wound within the previous year. Ethnic groups: African Americans, non-African Americans (White, Latino/Caribbean, Asian/Asian American, Arabic/Arabic American, or American Indian) | Indication of antibiotics, side effects, antibiotic use, antibiotic resistance. | Survey | Many respondents believed that antibiotics can cure colds and flu (10/26). More than half of the respondents (15/26) reported that antibiotics are good for treating infections caused by viruses. Nearly half of the respondents (10/26) also said that have few side effects and antibiotics do not hurt or harm people. Most people (23/26) said that it is not ok to use leftover antibiotics if you are sick or have a wound or sore. Similar number of respondents (22/26) also said that it is not OK to stop using antibiotic when a person starts feeling better and/or symptoms have stopped (eg, fever) and that antibiotics cannot be stored and used as needed at a later date. African American participants had significantly lower knowledge scores than non-African Americans.  A large number of respondents (69.2%) have heard of antibiotic resistance. Knowledge scores did not differ significantly by gender but higher antibiotic knowledge was strongly and significantly related to higher education. | 40% |
| Schuts 2019, The Netherlands | Non-western groups in Amsterdam insured with Achmea (from Surinamese, Ghanaian, Moroccan, and Turkish origin) and Dutch-born participants. | Indication antibiotics | Questionnaire | Non-Dutch groups were less knowledgeable about antibiotics than Dutch. Within the non-Dutch groups, the 1st generation were less knowledge than 2nd generation. Participants <25 had less knowledgeable than all other age groups. Women had more knowledge than men. Participants that requested antibiotic more had the lowest amount of knowledge. | 100% |
| Schwartz 2017, USA | Patients aged 18 and presenting at a participating clinic. Ethnic groups: Non-Hipanic/Latino, Hipanico/Latino; White only, Black only, American Indian/Alaska Native only, Asian/Pacific Islander only, Multiple/other race | Indication antibiotics, antibiotic use. | Survey | Majority of participants (87.3%) preferred avoiding antibiotics unless advised otherwise by their doctor. Most participants understood antibiotics are used to cure bacterial infections (37.9%), while a small proportion believed antibiotics are effective for viral infections (9.6%) or both (25.1%). Those who preferred to not take antibiotics were three times more likely to have correct knowledge about antibiotics compared to those who preferred taking them. White respondents were significantly more likely to have better antibiotic knowledge compared to any other race (Black, American Indian/ Alaska Native, Asian/ Pacific Islander, Multiple/ other race).  Women were 2 times more likely than men to have more knowledge about antibiotics. | 60% |
| Watkins 2015, USA | General population (i.e. all ethnic groups). Adult Hispanic consumers and health care providers. | Indication antibiotics, side effects, antibiotic use and antibiotic resistance. | Survey | Hispanic consumers were more likely to agree that when they have a cold, antibiotics prevent more serious illness (40% versus 17%) and help them get better more quickly (48% versus 25%). Knowledge of antibiotic side effect was generally comparable between Hispanic consumers and all consumers.  Hispanic consumers were less aware of potential dangers of antibiotic use, such as antibiotics becoming less effective after their use (antibiotic resistance) or that antibiotics might kill the “good” bacteria the body needs. | 60% |
| **Qualitative studies (n = 5)** | | | | | |
| Hika 2022, New Zealand | Indigenous Māori in New Zealand, who presented with symptoms of an upper respiratory tract infection (cold or flu symptoms). | Indication of antibiotics, antibiotic use. | Focus groups | Participants were not able to distinguish between a bacterial and viral infection and lacked knowledge about antibiotic usage. Participants expressed concerns about effect of antibiotics on their bodies. | 100% |
| Lescure 2022, the Netherlands | Community participants - 1st generation migrants from Turkey, Morocco, Suriname, Cape Verde, Syria and Dutch native. | Indication of antibiotics, side effects, antibiotic use | Focus groups | Some participants knew that antibiotics were only effective against bacterial infections and were aware of side effects (e.g. can induce sun sensitivity). Some participants thought that antibiotics were used for viral infections. Others felt that antibiotics weakened the body and reduced its ability to heal. A small number of people thought that antibiotics would create an adverse reaction in the body and would create antibodies against the antibiotics. Some participants were aware that antibiotics can also destroy healthy gut bacteria and several participants expressed an understanding of antibiotic resistance.  In general, study did not find noteworthy differences between immigrant and native Dutch participants. | 100% |
| Lindemeyer 2016, UK | Migrants who have been in the UK for >1 year but less than 5 years. Country of birth: Iran, Poland, Pakistan, India, Iraq, Somalia, Zimbabwe, Sudan, Cameroon, DR Congo, Uganda, Ivory Coast, China. | Indication antibiotics, side effects | Interviews | Participants from African countries and India spoke of the need to take antibiotics quickly to cure illness and prevent further infection. Participants from Iran and China and some African countries believed that antibiotics are ‘strong medicine’ with powerful effect on the individual, including strong side effects. | 100% |
| Westerling 2020, Germany, Sweden and The Netherlands | Turkish migrants living in Germany, Sweden, and the Netherlands over the age of 18. | Knoledge of antibiotic use | Focus groups and interviews | Reduced understanding of antibiotic rational use in Turkish participants compared to host population. Lower antibiotic knowledge linked with lower socioeconomic and educational level. | 100% |
| Whittaker 2019, Australia | Diverse ethnic communities from hospital in-patients, hospital interpreters and general public. ‘Diverse ethnic communities’ refers to first generation migrant communities in Australia, also often described as ‘culturally and linguistically diverse’ (CALD) communities. | Indication antibiotics, side effects/risks, antibiotic resistance, antibiotic use. | Interviews | Antibiotics considered strong and rapid medicines that potentially harm and weaken the body hence should be used sparingly. Home remedies preferred.  Mobile populations and travel contribute to AMR (contagion theory) also related to using/purchasing medicines from overseas.  Individual body becomes resistant; intolerant' body becomes inured to particular antibiotics (resistant bodies).  A number of informants were either vague or uncertain about exactly what antibiotics were and what they are used for.  Vague or lack of knowledge of AMR or use of antibiotics, even among patients infected with AMR infections. | 100% |
| **Mixed methods studies (n = 2)** | | | | | |
| Larson 2006, USA | Participants included (a) women of Latino origin, born either in the United States or elsewhere, but with Spanish as their first language; (b) members of households that included at least one preschool child; and (c) residents of northern Manhattan. | Indication antibiotics, risks, knowledge of antibiotic resistance | Focus group and questionnaires. | Most participants had taken antibiotics for symptoms suggestive of infection (fever, sore throat, ear infection), but others (5/15) also took for other unrelated symptoms (e.g. itching nausea). Majority thought that antibiotics could help cure a cold (56%) and  that antibiotics would help treating bacteria and viruses (56%). Some were aware that antibiotics can cause risks to health (36%).  Some thought that antibiotics should be stopped as soon as the person feels better (24%). Being able to identify antibiotics from a list of commonly used drugs varied considerably between participants (12-92%).  Many were aware that some germs are becoming harder to treat with antibiotics (44%) and that bacteria can become resistant to antibiotics if the dose (60%) or length (48%) of course is inadequate. | 20% |
| Norris 2009, New Zealand | Samoans living in New Zealand | Indication of antibiotics, identify antibiotics, antibiotic use. | Interviews and questionnaire | A minority of participants (2/112) were able to correctly answer the question of what antibiotics do. Just over half of the sample (54%) correctly identified antibiotics from a list of medicines; amoxicillin was identified correctly by the majority (81%), as welll as augmentin (80%). Majority (81%) believed antibiotics were useful for colds and flu and the majority also thought it would prevent cold and flus from getting worse (68%).  4. Majority (62%) thought antibiotics would help people get better sooner, relieve symptoms (57%) and some thought it would prevent serious illness (35%).  Some participants confused antibiotic with painkillers and other medications (such as allopurinol, epilepsy medication, topical NSAIDs) | 0% |
| *Hu 2014 would also fulfill inclusion criteria but sample same as Hu 2015 | | | | | |

**3. Detailed quality assessment of the included studies by study design using MMAT**

| **Quantitative descriptive design (n = 17)** | | | | | |
| --- | --- | --- | --- | --- | --- |
| **Study** | **Domains** | | | | |
|  | **Is the sampling strategy relevant to address the research question?** | **Is the sample representative of the target population?** | **Are the measurements appropriate?** | **Is the risk of nonresponse bias low?** | **Is the statistical analysis appropriate to answer the research question?** |
| Pieper 2020 | Y | NR | N | N | Y |
| Kuzujanakis 2003 | Y | Y | Y | N | Y |
| McNulty 2022 | Y | Y | Y | N | Y |
| Corbett 2005 | Y | NR | Y | NR | Y |
| Norris 2010 | N | NR | N | NR | Y |
| Watkins 2015 | Y | Y | NR | Y | N |
| McNulty 2019 | Y | Y | Y | NR | Y |
| Mason 2016 | Y | Y | N | NR | Y |
| Schwartz 2017 | Y | N | N | Y | Y |
| McNulty 2007 | Y | Y | Y | Y | Y |
| Alden 2006 | Y | NR | NR | N | Y |
| Broniatowski 2018 | Y | NR | Y | N | Y |
| Hu 2015 | Y | N | N | NR | Y |
| Panagakou 2012 | Y | Y | Y | Y | Y |
| Schuts 2019 | Y | Y | Y | Y | Y |
| Landers 2010 | Y | NR | N | Y | Y |
| Kandakai 1996 | Y | NR | N | Y | Y |
| **Qualitative design (n = 5 )** | | | | | |
|  | **Is the qualitative approach appropriate to answer the research question?** | **Are the qualitative data collection methods adequate to address the research question?** | **Are the findings adequately derived from the data?** | **Is the interpretation of results sufficiently substantiated by data?** | **Is there coherence between qualitative data sources, collection, analysis and interpretation?** |
| Lindemeyer 2016 | Y | Y | Y | Y | Y |
| Whittaker 2019 | Y | Y | Y | Y | Y |
| Hika 2022 | Y | Y | Y | Y | Y |
| Westerling 2020 | Y | Y | Y | Y | Y |
| Lescure 2022 | Y | Y | Y | Y | Y |
| **Mixed-method design (n = 2)*** | | | | | |
| Larson 2006 | **Is the qualitative approach appropriate to answer the research question?** | **Are the qualitative data collection methods adequate to address the research question?** | **Are the findings adequately derived from the data?** | **Is the interpretation of results sufficiently substantiated by data?** | **Is there coherence between qualitative data sources, collection, analysis and interpretation?** |
|  | Y | Y | NR | Y | Y |
|  | **Is the sampling strategy relevant to address the research question?** | **Is the sample representative of the target population?** | **Are the measurements appropriate?** | **Is the risk of nonresponse bias low?** | **Is the statistical analysis appropriate to answer the research question?** |
|  | N | N | N | NR | Y |
|  | **Is there an adequate rationale for using a mixed methods design to address the research question?** | **Are the different components of the study effectively integrated to answer the research question?** | **Are the outputs of the integration of qualitative and quantitative components adequately interpreted?** | **Are divergences and inconsistencies between quantitative and qualitative results adequately addressed?** | **Do the different components of the study adhere to the quality criteria of each tradition of the methods involved?** |
|  | NR | Y | Y | NR | Y |
| Norris 2009 | **Is the qualitative approach appropriate to answer the research question?** | **Are the qualitative data collection methods adequate to address the research question?** | **Are the findings adequately derived from the data?** | **Is the interpretation of results sufficiently substantiated by data?** | **Is there coherence between qualitative data sources, collection, analysis and interpretation?** |
|  | Y | Y | NR | NR | NR |
|  | **Is the sampling strategy relevant to address the research question?** | **Is the sample representative of the target population?** | **Are the measurements appropriate?** | **Is the risk of nonresponse bias low?** | **Is the statistical analysis appropriate to answer the research question?** |
|  | NR | NR | N | NR | **N** |
|  | **Is there an adequate rationale for using a mixed methods design to address the research question?** | **Are the different components of the study effectively integrated to answer the research question?** | **Are the outputs of the integration of qualitative and quantitative components adequately interpreted?** | **Are divergences and inconsistencies between quantitative and qualitative results adequately addressed?** | **Do the different components of the study adhere to the quality criteria of each tradition of the methods involved?** |
|  | N | N | Y | NR | N |

Y: Yes; N: No; NR: Not Reported

^*^ In each study, the risk of bias was assessed separately for quantitative and qualitative components, and for the mixed method component. The overall MMAT score was based on the ‘yes’ responses in the lowest rated component

**4) List of excluded studies at full-text screening stage with reasons for exclusion**

|  | **First Author** | **Title** | **Journal** | **Year of publication** | **Reason for exclusion** |
| --- | --- | --- | --- | --- | --- |
| 1 | Goggin K | Reductions in Parent Interest in Receiving Antibiotics following a 90-Second Video Intervention in Outpatient Pediatric Clinics | Journal of pediatrics - Volume 225, Issue 0, pp. 138‐145 | 2020 | Wrong outcome |
| 2 | Wilding, S | Development and randomized controlled trial of an animated film aimed at reducing behaviours for acquiring antibiotics | Jac-antimicrobial resistance - Volume 3, Issue 2 | 2021 | Wrong outcome |
| 3 | Medina-Perucha, L | Autonomy, power dynamics and antibiotic use in primary healthcare: a qualitative study | PloS one - Volume 15, Issue 12, pp. e0244432 | 2020 | Wrong outcome |
| 4 | Black, M | Attitudes and awareness of Australian women regarding peripartum antibiotic use: A multicentre survey. | Australian & New Zealand Journal of Obstetrics & Gynaecology - Volume 63, Issue 2, pp. 171-177 | 2023 | Wrong outcome |
| 5 | Alkirawan, R | Perspectives of Syrian refugees on antibiotic use and prescribing in Dutch primary care: a qualitative study. | International Journal of Migration, Health & Social Care - Volume 18, Issue 2, pp. 153-163 | 2022 | Wrong outcome |
| 6 | Khazen, M | 'Nesef Doctora'—When mothers are considered to be 'half‐doctors': Self‐medication with antibiotics and gender roles in the Arab society in Israel. | Sociology of Health & Illness - Volume 43, Issue 2, pp. 408-423 | 2021 | Wrong outcome |
| 7 | Madden H | "Always paracetamol, they give them paracetamol for everything": a qualitative study examining Eastern European migrants' experiences of the UK health service. | BMC Health Services Research - Volume 17, Issue 0, pp. 1-10 | 2017 | Wrong outcome |
| 8 | Jie Hu, Z | Non-prescribed antibiotic use and general practitioner service utilisation among Chinese migrants in Australia. | Australian Journal of Primary Health - Volume 22, Issue 5, pp. 434-439 | 2016 | Wrong outcome |
| 9 | Migault, C | Poor knowledge among French travellers of the risk of acquiring multidrug-resistant bacteria during travel. | Journal of Travel Medicine - Volume 24, Issue 1, pp. N.PAG-N.PAG | 2016 | Wrong outcome |
| 10 | Wiklund, S | Knowledge and understanding of antibiotic resistance and the risk of becoming a carrier when travelling abroad: A qualitative study of Swedish travellers. | Scandinavian Journal of Public Health - Volume 43, Issue 3, pp. 302-308 | 2015 | Wrong outcome |
| 11 | Broniatowski D | Germs Are Germs, and Why Not Take a Risk? Patients’ Expectations for Prescribing Antibiotics in an Inner-City Emergency Department. | Medical Decision Making - Volume 35, Issue 1, pp. 60-67 | 2015 | Wrong population |
| 12 | Stockwell MS | Improving Care of Upper Respiratory Infections Among Latino Early Head Start Parents. | Journal of Immigrant & Minority Health - Volume 12, Issue 6, pp. 925-931 | 2010 | Wrong population |
| 13 | Mainous AG | Factors affecting Latino adults' use of antibiotics for self-medication. | Journal of the American Board of Family Medicine - Volume 21, Issue 2, pp. 128-134 | 2008 | Wrong outcome |
| 14 | Alder SC | Reducing parental demand for antibiotics by promoting communication skills. | American Journal of Health Education - Volume 36, Issue 3, pp. 132-147 | 2005 | Wrong outcome |
| 15 | Mangione-Smith R | Racial/ethnic variation in parent expectations for antibiotics: implications for public health campaigns. | Pediatrics - Volume 113, Issue 0, pp. e385-94 | 2004 | Wrong outcome |
| 16 | Edwards DJ | Parental use and misuse of antibiotics: are there differences in urban vs. suburban settings? | Academic Emergency Medicine - Volume 9, Issue 1, pp. 22-26 | 2002 | Wrong outcome |
| 17 | Alexa E.-A | A European questionnaire-based study on population awareness and risk perception of antimicrobial resistance | FEMS Microbiology Letters - Volume 366, Issue 17, pp. fnz221 | 2019 | Wrong population |
| 18 | Wardley A | Would men who have sex with men support less frequent screening for asymptomatic chlamydia and gonorrhoea to improve antibiotic stewardship? A qualitative study | Sexual health - Volume 20, Issue 2, pp. 148-157 | 2023 | Wrong outcome |
| 19 | Pallos P | HSD37 Information Seeking Behaviour of the Hungarian Population Regarding Antibiotics and Common Infectious Ailments: An Infodemiological and Thematic Analysis Using Google TRENDSTM | Value in Health - Volume 25, Issue 12, pp. S280-S281 | 2022 | Wrong population |
| 20 | Anderson R | A nationwide parent survey of antibiotic use in Australian children | The Journal of antimicrobial chemotherapy - Volume 0, Issue 0 | 2020 | Wrong population |
| 21 | Seipel M.B.A | Patient Knowledge and Experiences With Antibiotic Use and Delayed Antibiotic Prescribing in the Outpatient Setting | Journal of pharmacy practice - Volume 0, Issue 0, | 2019 | Wrong population |
| 22 | Hoffmann K | Oral contraceptives and antibiotics. A cross-sectional study about patients' knowledge in general practice | Reproductive Health - Volume 0, Issue 0 | 2015 | Wrong outcome |
| 23 | Munthe C | Non-prescription acquisition of antibiotics: Prevalence, motives, pathways and explanatory factors in the Swedish population | PLoS ONE - Volume 17, Issue 9, pp. e0273117 | 2022 | Wrong outcome |
| 24 | Renny M.H | Caregiver Practices and Knowledge Regarding Leftover Prescription Medications in Homes With Children | Pediatric Emergency Care - Volume 38, Issue 9, pp. E1557-E1563 | 2022 | Wrong outcome |
| 25 | David J.-C | Knowledge and perceptions of antibiotic resistance in the French population | Infectious Diseases Now - Volume 52, Issue 5, pp. 306-310 | 2022 | Wrong outcome |
| 26 | Guo H | The Associations between Poor Antibiotic and Antimicrobial Resistance Knowledge and Inappropriate Antibiotic Use in the General Population Are Modified by Age | Antibiotics - Volume 11, Issue 1, pp. 47 | 2022 | Wrong population |
| 27 | Black M | Attitudes toward antibiotic use in pregnancy and after birth - A survey of Australian women | Obstetric Medicine - Volume 15, Issue 1, pp. 26 | 2022 | Wrong publication type |
| 28 | Abar C.C | Parenting, health, and use of medications among college youth: The PHARMACY survey study | Preventive Medicine Reports - Volume 24, Issue 0, pp. 101623 | 2021 | Wrong outcome |
| 29 | Ancillotti M | An Effort Worth Making: A Qualitative Study of How Swedes Respond to Antibiotic Resistance | Public Health Ethics - Volume 14, Issue 1, pp. 1-11 | 2021 | Wrong outcome |
| 30 | Morrell L | Public preferences for delayed or immediate antibiotic prescriptions in UK primary care: A choice experiment | PLoS Medicine - Volume 18, Issue 8, pp. e1003737 | 2021 | Wrong population |
| 31 | Newbigging-Lister A | Knowledge, use and acceptability of antibiotics to prevent sexually transmitted infections among respondents of the 2020 PrEP User Survey | HIV Medicine - Volume 22, Issue 0, pp. 45 | 2021 | Wrong publication type |
| 32 | Van Hecke O | Using evidence-based infographics to increase parents' understanding about antibiotic use and antibiotic resistance: A proof-of-concept study | JAC-Antimicrobial Resistance - Volume 2, Issue 4, pp. dlaa102 | 2020 | Wrong population |
| 33 | Hughes G. | Evaluating patient attitudes to increased patient engagement with antimicrobial stewardship: A quantitative survey | JAC-Antimicrobial Resistance - Volume 2, Issue 3 | 2020 | Wrong outcome |
| 34 | Bianco A | Knowledge and practices regarding antibiotics use | Evolution, Medicine and Public Health - Volume 2020, Issue 1, pp. 129-138 | 2020 | Wrong population |
| 35 | Ancillotti M | Preferences regarding antibiotic treatment and the role of antibiotic resistance: A discrete choice experiment | International Journal of Antimicrobial Agents - Volume 56, Issue 6, pp. 106198 | 2020 | Wrong outcome |
| 36 | Worthington A.K | Perceptions of Responsibility for Antibiotic Resistance: Implications for Stewardship Campaigns | Journal of health communication - Volume 25, Issue 9, pp. 703-711 | 2020 | Wrong outcome |
| 37 | Hansen M | Symptoms and situations predispose patients to use antibiotics without medical advice | Open Forum Infectious Diseases - Volume 7, Issue 0, pp. S200 | 2020 | Wrong publication type |
| 38 | Minejima E | Understanding patient perceptions and attitudes toward urinary tract infections and treatment in a medically underserved population | JACCP Journal of the American College of Clinical Pharmacy - Volume 2, Issue 6, pp. 616-622 | 2019 | Wrong outcome |
| 39 | Hernandez-Diaz I | Knowledge and beliefs, behaviors, and adherence among Latino parents or legal guardians related to antibiotic use for upper respiratory tract infections in children under 6 years of age | Journal of the American Pharmacists Association - Volume 59, Issue 4, pp. 506-513 | 2019 | Wrong population |
| 40 | Goggin K | Reductions in parent interest for antibiotics following a two minute tablet delivered intervention in pediatric outpatient clinics | Pediatrics - Volume 144, Issue 2, pp. - published 2019-01-01 | 2019 | Wrong publication type |
| 41 | Pieper B | Knowledge, attitudes, beliefs, and behaviors about antibiotics by persons with wounds | Wound Repair and Regeneration - Volume 27, Issue 3, pp. A16 | 2019 | Wrong publication type |
| 42 | Cabral C | Understanding the influence of parent-clinician communication on antibiotic prescribing for children with respiratory tract infections in primary care: a qualitative observational study using a conversation analysis approach | BMC family practice - Volume 20, Issue 1, pp. 102 | 2019 | Wrong outcome |
| 43 | Dekker A.R.J | Parents' attitudes and views regarding antibiotics in the management of respiratory tract infections in children: A qualitative study of the influence of an information booklet | BJGP Open - Volume 2, Issue 2, | 2018 | Wrong outcome |
| 44 | Navaro M | Knowledge, attitudes, and practice regarding medication use in pregnant women in Southern Italy | PLoS ONE - Volume 13, Issue 6, pp. e0198618 | 2018 | Wrong outcome |
| 45 | Kamata K | Public knowledge and perception about antimicrobials and antimicrobial resistance in Japan: A national questionnaire survey in 2017 | PLoS ONE - Volume 13, Issue 11, pp. e0207017 | 2018 | Wrong population |
| 46 | Lee C.H.J | Demographic and psychological factors associated with feelings of antibiotic entitlement in New Zealand | Antibiotics - Volume 7, Issue 3, pp. 82 | 2018 | Wrong outcome |
| 47 | Pouwels K | Awareness of appropriate antibiotic use in primary care for influenza-like illness: Evidence of improvement from UK population-based surveys | Antibiotics - Volume 9, Issue 10, pp. 1-16 | 2020 | Wrong population |
| 48 | Akici A | Patients' attitudes and knowledge about drug use: A survey in Turkish family healthcare centres and state hospitals | Turkish Journal of Medical Sciences - Volume 47, Issue 5, pp. 1472-1481 | 2017 | Wrong outcome |
| 49 | Gaygisiz U | Socio-economic factors, cultural values, national personality and antibiotics use: A cross-cultural study among European countries | Journal of Infection and Public Health - Volume 10, Issue 6, pp. 755-760 | 2017 | Wrong outcome |
| 50 | Mazinska B | Surveys of public knowledge and attitudes with regard to antibiotics in Poland: Did the European Antibiotic Awareness Day campaigns change attitudes? | PLoS ONE - Volume 12, Issue 2, pp. e0172146 | 2017 | Wrong population |
| 51 | Freidoony L | From Visiting a Physician to Expecting Antibiotics: Korean Perspectives and Practices toward Respiratory Tract Infections | Journal of Korean medical science - Volume 32, Issue 2, pp. 278-286 | 2017 | Wrong outcome |
| 52 | Beyene K | Prescription medicine sharing: Exploring patients' beliefs and experiences | Journal of Pharmaceutical Policy and Practice - Volume 9, Issue 1, pp. 23 | 2016 | Wrong outcome |
| 53 | Marques C.F | Analysis of behaviours and attitudes of antibiotics use in Algarve's population | International Journal of Clinical Pharmacy - Volume 38, Issue 6, pp. 563 | 2016 | Wrong publication type |
| 54 | Cabral C | Influence of Clinical Communication on Parents' Antibiotic Expectations for Children With Respiratory Tract Infections | Annals of family medicine - Volume 14, Issue 2, pp. 141-147 | 2016 | Wrong outcome |
| 55 | Drozd M | Knowledge, attitude and perception regarding antibiotics among polish patients | Acta Poloniae Pharmaceutica - Drug Research - Volume 72, Issue 4, pp. 807-817 | 2015 | Wrong population |
| 56 | Hu J | Bringing antibiotics from overseas and self-medication amongst Australian Chinese migrants | Internet Journal of Infectious Diseases - Volume 14, Issue 1 | 2015 | Wrong outcome |
| 57 | Martin-Perez M | Predictors of medication use in the Roma population in Spain: A population-based national study | Public Health - Volume 129, Issue 5, pp. 453-459 | 2015 | Wrong outcome |
| 58 | Branch-Elliman W | Preliminary outcomes from an educational intervention to promote antimicrobial stewardship and improve the care of older adults with infections | Journal of the American Geriatrics Society - Volume 63, Issue 0, pp. S54 | 2015 | Wrong publication type |
| 59 | Azeem M | Knowledge, attitude and practice (KAP) survey concerning antimicrobial use among Australian Hajj pilgrims | Infectious Disorders - Drug Targets - Volume 14, Issue 2, pp. 125-132 | 2014 | Wrong population |
| 60 | Ramalhinho I | Assessing determinants of self-medication with antibiotics among Portuguese people in the Algarve Region | International Journal of Clinical Pharmacy - Volume 36, Issue 5, pp. 1039-1047 | 2014 | Wrong outcome |
| 61 | Rivers P | Perceptions of antibiotic use and microbial resistance-a pilot study to test the potential of a fictitious vignette to assess lay beliefs and attitudes | International Journal of Pharmacy Practice - Volume 22, Issue 0, pp. 55-56 | 2014 | Wrong outcome |
| 62 | Norris P | Public beliefs about antibiotics, infection and resistance: A qualitative study | Antibiotics - Volume 2, Issue 4, pp. 465-476 | 2013 | Wrong population |
| 63 | Horton S | Reasons for self-medication and perceptions of risk among Mexican migrant farm workers | Journal of immigrant and minority health / Center for Minority Public Health - Volume 14, Issue 4, pp. 664-672 | 2012 | Wrong outcome |
| 64 | Dunn-Navarra A.M | Parental health literacy, knowledge and beliefs regarding upper respiratory infections (URI) in an urban Latino immigrant population | Journal of urban health : bulletin of the New York Academy of Medicine - Volume 89, Issue 5, pp. 848-860 | 2012 | Wrong outcome |
| 65 | McNulty C A M | The English antibiotic awareness campaigns: Did they change the public's knowledge of and attitudes to antibiotic use? | Journal of Antimicrobial Chemotherapy - Volume 65, Issue 7, pp. 1526-1533 | 2010 | Wrong population |
| 66 | Mainous III A G | A community intervention to decrease antibiotics used for self-medication among latino adults | Annals of Family Medicine - Volume 7, Issue 6, pp. 520-526 | 2009 | Wrong outcome |
| 67 | Croft D R | Impact of a child care educational intervention on parent knowledge about appropriate antibiotic use | Wisconsin Medical Journal - Volume 106, Issue 2, pp. 78-84 | 2007 | Wrong outcome |
| 68 | Soma M | Patients' expectations of antibiotics for acute respiratory tract infections (ARTI): A questionnaire survey at the Oslo emergency centre, winter 2002-2003 | Tidsskrift for den Norske Laegeforening - Volume 125, Issue 15, pp. 1994-1997 | 2005 | Not in English |
| 69 | Bunuel Alvarez J C | Antibiotic use in primary care. Do we know what parents think? | Anales de Pediatria - Volume 61, Issue 4, pp. 298-304 | 2004 | Not in English |
| 70 | Larson E | Antibiotic use in Hispanic households, New York City | Emerging Infectious Diseases - Volume 9, Issue 9, pp. 1096-1102 | 2003 | Wrong outcome |
| 71 | Britten N | Patients' attitudes to medicines and expectations for prescriptions | Health Expectations - Volume 5, Issue 3, pp. 256-269 | 2002 | Wrong outcome |
| 72 | Deschepper R | Cross-cultural differences in lay attitudes and utilisation of antibiotics in a Belgian and a Dutch city | Patient Education and Counseling - Volume 48, Issue 2, pp. 161-169 | 2002 | Wrong outcome |
| 73 | Hu, J | In-home antibiotic storage among Australian Chinese migrants. | International journal of infectious diseases : IJID : official publication of the International Society for Infectious Diseases - Volume 26, Issue 0, pp. 103-6 | 2014 | Duplicate sample |
| 74 | Genne D | Antibiotics and some popular beliefs | Revue Medicale de la Suisse Romande - Volume 118, Issue 3, pp. 221-225 | 1998 | Not in English |
| 75 | Laytner L | Perspectives on Non-Prescription Antibiotic Use among Hispanic Patients in the Houston Metroplex. | Journal of the American Board of Family Medicine : JABFM - Volume 0, Issue 101256526, | 2023 | Wrong outcome |
| 76 | Sager S | Assessment of the changing trends in maternal knowledge about management of fever and antibiotic use in the last decade in Turkiye. | The Turkish journal of pediatrics - Volume 65, Issue 2, pp. 269-277 | 2023 | Wrong population |
| 77 | Vanbaelen T | Prophylactic use of antibiotics for sexually transmitted infections: awareness and use among HIV PrEP users in Belgium. | Sexually transmitted infections - Volume 98, Issue 8, pp. 625 | 2022 | Wrong outcome |
| 78 | Alsaleh R | The Awareness of Pregnant Patient about Effect of Antibiotics in Pregnancy. | Journal of microscopy and ultrastructure - Volume 7, Issue 2, pp. 72-77 | 2019 | Wrong population |
| 79 | McNulty C | Delayed/back up antibiotic prescriptions: what do the public think? | BMJ open - Volume 5, Issue 11, pp. e009748 | 2015 | Wrong outcome |
| 80 | Alden D | Investigating approaches to improving appropriate antibiotic use among higher risk ethnic groups. | Hawaii medical journal - Volume 69, Issue 11, pp. 260-3 | 2010 | Wrong outcome |
| 81 | Faber M | Antibiotics for the common cold: expectations of Germany's general population. | Euro surveillance : bulletin Europeen sur les maladies transmissibles = European communicable disease bulletin - Volume 15, Issue 35 | 2010 | Wrong population |
| 82 | White D | Parental satisfaction without antibiotics | The Nurse practitioner - Volume 35, Issue 3, pp. 40-2 | 2010 | Wrong outcome |
| 83 | Radosevic N | Attitudes towards antimicrobial drugs among general population in Croatia, Fyrom, Greece, Hungary, Serbia and Slovenia. | Pharmacoepidemiology and drug safety - Volume 18, Issue 8, pp. 691-6 | 2009 | Wrong population |
| 84 | Larson E | Knowledge and misconceptions regarding upper respiratory infections and influenza among urban Hispanic households: need for targeted messaging. | Journal of immigrant and minority health - Volume 11, Issue 2, pp. 71-82 | 2009 | Wrong outcome |
| 85 | Deschepper R | Are cultural dimensions relevant for explaining cross-national differences in antibiotic use in Europe? | BMC health services research - Volume 8, Issue 101088677, pp. 123 | 2008 | Wrong outcome |
| 86 | Grigoryan L | Determinants of self-medication with antibiotics in Europe: the impact of beliefs, country wealth and the healthcare system. | The Journal of antimicrobial chemotherapy - Volume 61, Issue 5, pp. 1172-9 | 2008 | Wrong outcome |
| 87 | Grigoryan L | Attitudes, beliefs and knowledge concerning antibiotic use and self-medication: a comparative European study. | Pharmacoepidemiology and drug safety - Volume 16, Issue 11, pp. 1234-43 | 2007 | Wrong outcome |
| 88 | Vaananen M | Self-medication with antibiotics--does it really happen in Europe? | Health policy (Amsterdam, Netherlands) - Volume 77, Issue 2, pp. 166-71 | 2006 | Wrong outcome |
| 89 | Emslie M | Public knowledge, attitudes and behaviour regarding antibiotics--a survey of patients in general practice. | The European journal of general practice - Volume 9, Issue 3, pp. 84-90 | 2003 | Wrong outcome |
| 90 | McKee M | Antibiotic use for the treatment of upper respiratory infections in a diverse community. | The Journal of family practice - Volume 48, Issue 12, pp. 993-6 | 1999 | Wrong outcome |

**4. Example of data analysis process**
